# Obese parents – obese children? Psychological-psychiatric risk factors of parental behavior and experience for the development of obesity in children aged 0–3: study protocol

**DOI:** 10.1186/1471-2458-13-1193

**Published:** 2013-12-17

**Authors:** Matthias Grube, Sarah Bergmann, Anja Keitel, Katharina Herfurth-Majstorovic, Verena Wendt, Kai von Klitzing, Annette M Klein

**Affiliations:** 1 Integrated Research and Treatment Center (IFB) AdiposityDiseases, University of Leipzig, Leipzig, Germany; 2 Department of Child and Adolescent Psychiatry, Psychotherapy, and Psychosomatics, University of Leipzig, Leipzig, Germany

**Keywords:** Overweight, Obesity, Family, Interaction, Early investigation

## Abstract

**Background:**

The incidences of childhood overweight and obesity have increased substantially and with them the prevalence of associated somatic and psychiatric health problems. Therefore, it is important to identify modifiable risk factors for early childhood overweight in order to develop effective prevention or intervention programs. Besides biological factors, familial interactions and parental behavioral patterns may influence children’s weight development. Longitudinal investigation of children at overweight risk could help to detect significant risk and protective factors. We aim to describe infants’ weight development over time and identify risk and protective factors for the incidence of childhood obesity. Based on our findings we will draw up a risk model that will lay the foundation for an intervention/prevention program.

**Methods/Design:**

We present the protocol of a prospective longitudinal study in which we investigate families with children aged from 6 months to 47 months. In half of the families at least one parent is obese (risk group), in the other half both parents are normal weight (control group). Based on developmental and health-psychological models, we consider measurements at three levels: the child, the parents and parent–child-relationship. Three assessment points are approximately one year apart. At each assessment point we evaluate the psychological, social, and behavioral situation of the parents as well as the physical and psychosocial development of the child. Parents are interviewed, fill in questionnaires, and take part in standardized interaction tasks with their child in a feeding and in a playing context in our research laboratory. The quality of these video-taped parent–child interactions is assessed by analyzing them with standardized, validated instruments according to scientific standards.

**Discussion:**

Strengths of the presented study are the prospective longitudinal design, the multi-informant approach, including the fathers, and the observation of parent–child interaction. A limitation is the variation in children’s age.

## Background

The incidence of childhood overweight has increased substantially [1,2]. Eventually, this is likely to result in increases of adult overweight and obesity together with the consequent health problems associated with it. Although not all cases of adult obesity begin in childhood, there is a considerable proportion that does [3-5]. In addition, prevalence rates for overweight and obesity in early childhood have increased. Therefore, it is important to identify modifiable risk factors for early childhood overweight in order to develop effective prevention or intervention programs. This is the main aim of our prospective longitudinal study which is described in detail in the present study protocol. In the following, epidemiology of obesity and overweight, weight development in the first three years of life and risk factors for developing overweight/obesity are described.

### Epidemiology of obesity

The prevalence of overweight and obesity among adults and children has been identified as an epidemic and as one of the leading health indicators for communities [6]. For example, in the past 30 years, the prevalence of overweight has more than doubled among 2–5 year-olds (5–12.4% >95th body mass index percentile) [1,2]. Notwithstanding research suggesting that overweight and obesity trends are leveling off [2,7] the proportion of pediatric populations classified as obese still remains very large [8]. In Germany and in other European countries overweight in children and adolescents is a significant health problem and its incidence has increased alarmingly [9,10]. In addition to the physical consequences, mental health problems and victimization are associated with obesity in children and adolescents [11,12].

### Weight development in the first three years of life

According to the WHO definition the Body Mass Index (BMI) is calculated as a person’s weight in kilograms divided by the square of the person’s height in meters (kg/m^2^) [13]. The BMI is the most commonly used clinical method of assessing underweight, overweight and obesity in children and youth and monitoring weight status throughout maturation. Its use is recommended by the Childhood Group of the International Obesity Task Force (IOTF) and the European Childhood Obesity Group (ECOG) [14,15]. Age- and sex-specific BMI growth charts are used for diagnosing and tracking overweight and obesity. In Germany, a BMI reference data set was compiled from 17 different regional studies including 17,147 boys and 17,275 girls aged 0–18 years [16]. Normal weight development is characterized by rapid weight gain in the first year of life and slower weight gain later on. A first maximum at the age of 12 months follows a decline. Whereas normal weight children reach the BMI value of the 12 months maximum again at the age of 9–10 years, overweight and obese children reach it much earlier (age 5–7 years). It is important to assess weight development at birth, as higher birth weight and rapid weight gain in the first 4 months of life are risk factors for a later overweight both in children and adults [17-19].

### Genetic and psychosocial etiology of obesity

Many reports have addressed the question, whether there is a genetic component to individual differences observed in body weight and obesity during childhood. For example, Stunkard and colleagues studied samples of identical and fraternal twins, either reared apart or reared together, and concluded that genetic influences on BMI are substantial, whereas childhood environment has a smaller influence [20,21]. The estimated heritability was between 60% and 70% with the remaining variance attributable to nonshared environment. Dubois et al. reported even higher heritability estimates. They analyzed data from the Quebec Newborn Twin Study (QNTS), including 177 twin pairs and reported heritability estimates ranging between 84% and 88% [22]. Similar high heritability estimates were found in a study by Demerath and colleagues investigating 501 white infants in 164 nuclear and extended families in the Fels Longitudinal Study [23]. The data showed that additive genetic effects explained a high proportion of the variance in infant body weight, increasing from 65% at 12 months to 88% at 24 months and to 95% at 36 months. Genetic influence relates to metabolism and to behavior, both of which influence energy uptake and use [24].

Genome wide association studies have identified several loci for obesity-related phenotypes. A genome wide study in 249,796 individuals revealed a total of 32 loci associated with BMI [25]. In spite of these advances, the identified loci explain only 1.45% of the variance in BMI, or 2-4% of genetic variance based on an estimated heritability of 40-70%. Parental obesity is an important risk factor which contributes both genetic and family environmental influences for childhood overweight [3,17,21,26]–[28]. Results of a study by Jacobson et al. agree with the hypothesis that assortative mating for obesity confers a higher risk of obesity in the offspring generation. They conclude that parental obesity concordance is a strong, easily identifiable genetic risk factor that should be considered in research and primary prevention programs [29].

### Activity, diet, lifestyles

The term “lifestyle” is much discussed in connection with obesity [30,31]. Activities described as lifestyle factors include doing sports [32,33], television consumption [34], or the use of computer games [35,36] among others. Dietary behavior is generally also subsumed under lifestyle. In the first months of life, the form of nutrition parents choose seems to be important for the child’s weight development. A lot of studies show that breastfeeding is a preventive factor for weight development, while early weaning promotes weight gain [37,38]. However, other studies did not find these effects [37]. In a meta-analysis, Arenz et al. came to the conclusion that breastfeeding seems to have a small but consistent protective effect against obesity in children [39]. Parents largely determine the availability of healthy and unhealthy foods in the home [40]. Especially for young children, the family provides the environment in which patterns of food intake and activity are learned thus shaping children’s eating habits [3,40,41]. The relationship of activity and obesity in childhood has been investigated with inconsistent findings [3,42], although some studies have found a modest relation [43-45].

### Child attributes and parental behavior

In a prospective study of 150 children from birth to 9.5 years of age, Agras and colleagues assessed multiple hypothesized risk factors for childhood overweight [3]. Analyses identified five risk factors: parental overweight, child temperament, tantrums over food, low parental concerns about their child’s thinness and less hours of sleep at ages 3 to 4. The strongest direct effect on childhood overweight was parental overweight, which was additionally mediated by the child’s temperament with a combination of approach and impulsivity leading to a predisposition. As a possible mechanism they discussed that overweight parents may overcontrol the behavior of such children [46,47], for example by prompting food use excessively which in turn may increase caloric intake, as shown in laboratory studies [48]–[50]. Hours of sleep were negatively related with overweight, consistent with earlier findings [51]. The risk factor of low parental concern about child’s thinness might result from parental preference for a slim child coupled with an avid feeding pattern of the child which might lead to overcontrol of the child’s feeding behaviors. This, in turn, may serve to disrupt the child’s learning of self-control thus furthering the effects of the avid feeding pattern [3]. Also Graziano and colleagues reported an association between self-regulation skills of toddlers and the risk for overweight at 5.5 years of age even after controlling for BMI at 2 years of age [52]. Moreover, there is an association between the ability to delay gratification of 4-year-olds and their BMI 30 years later [53]. Regarding the parent–child relationship, Anderson and colleagues found that a poor mother-child relationship followed by an insecure mother-child attachment in early childhood is associated with a higher prevalence of obesity in adolescence [54].

### Psychosocial strain and stress

Low socioeconomic status (SES) is strongly associated with overweight and obesity and mental health of children [55,56]. Childhood SES is inversely related to overweight and obesity [26] and has long-lasting impact also on adult obesity [56]. Moreover, results of longitudinal studies have shown stressful life events and low quality of family relationships to increase the risk of psychopathological developments in children more generally [57-59]. The contribution of environmental factors (which include psychosocial strain) to BMI development is estimated at 10-40% [24]. Diverse care situations or varying infant and toddler temperaments can affect weight differently [37]. Moreover, the parents’ experience of stress after the birth of the child and during toddlerhood affects the development of the child [60]. The connections between eating and coping with stress have been documented [61,62]. Eating despite lack of hunger, in order to satisfy other needs, had no effects on the BMI of daughters of normal weight mothers but did affect the BMI of daughters of overweight mothers [63]. Furthermore, former research indicates, that patients with eating disorders (anorexia, bulimia) have problems with experiencing, describing and identifying their own emotions which implicates an association with the construct of alexithymia. Some studies implicate a relationship between obesity and alexithymic characteristics [64-66] whereas others don’t [67]. Research not only suggests that obese subjects have a diminished ability to decode nonverbal expressions of emotions, but that mothers of children with an early onset of obesity have an insufficient capacity to decode nonverbal expressions of emotions [68,69].

### Psychiatric comorbidity and depression

Various authors indicate connections between increasing weight and psychiatric comorbidity [70,71]. Depressive symptoms, suicidality, dissatisfaction with one’s own body, and low self-confidence occur more frequently in overweight and obese patients [72-77]. However, there are also studies that found negligible connections or none at all [77], or where gender-specific characteristics were identified, e.g. depressive symptoms were more common among obese primary-school girls compared to boys [70,78]. In a study in Switzerland mental disorders were found in 39% of obese children aged 8–12 of a referred clinical sample. Moreover, 10–12 year old overweight children of a representative study sample had significantly higher scores regarding social problems and dissocial behavior as indexed by the CBCL compared to their normal weight peers [79].

### Aims

We aim at describing changes in infants’ weight over time and identifying risk and protective factors for the occurrence of childhood overweight and obesity that involve biological as well as psychological factors occurring prenatally or in the first three years of life. Based on developmental and health-psychological models of developmental psychology and developmental psychopathology, we consider biological and psychological characteristics on three levels:

a) the level of the child’s individual psychological and biological functioning

b) the level of the parental functioning, and

c) the level of the family and parent–child interaction.

At the *level of the child’s individual functioning,* we focus on physical and mental development expecting higher BMI percentiles and higher internalizing and externalizing symptoms in the risk group. At the *level of the parental functioning*, we expect obesity-typical problems with respect to the parents’ resources in reacting to pressure and stress. At the* level of the family and parent–child interaction*, we hypothesize that obese parents react differently from non-obese parents in interactions with their children, in particular in infancy and early childhood. We assume that they tend to respond to their children less actively, more passively, or resort more quickly to offering food if their child shows distress. The compensation mechanism of increased food intake as a reaction to stress and psychic upset is known to play a role in obesity. Since there is a lack of data in this field, we consider it important to analyze the special risk factors that obese parents “bring to” the parent–child relationship.

Starting from these considerations, we focus on four parental factors and their effects on the children’s weight and behavioral development:

a) parental attitudes to feeding and nutrition,

b) parental psychological symptoms, in particular parents’ or mothers’ depression,

c) parenting stress, and

d) parental behavior and lifestyles

As relevant childhood factors we consider the temperament and innate regulatory ability of the child. Moreover, we investigate parent–child interactions in two different contexts (playing and feeding). As outcome we assess the trajectories of the child’s weight development, especially the development of overweight and obesity. We hypothesize that fundamental characteristics of the behavior of obese children are already established during the first three years of life through early learning, since experiences that are implicitly relevant in further development are collected and set the pattern for the handling of food and needs.

Specifically, we pursue the following research objectives:

1) Describing changes in children’s weight over time and to model increases, decreases, or stability in weight patterns longitudinally

2) Assessing typical phenomenological characteristics of the biological and psychological development of children in families with at least one obese parent compared to children in families with normal weight parents

3) Identifying characteristics of parental behavior and attitudes, which are risk or protective factors for the development of childhood obesity

4) Assessing possible characteristics of the parent–child interaction

5) Investigating possible interactions between psychological, social and biological risk factors

6) Using the results and risk factors identified to draw up a proposals for an intervention program for obese parents and their toddlers

## Methods/Design

### Research design and sample

We conduct a prospective longitudinal study with three assessment points (t1, t2, and t3), each assessment point being 11 months apart. The sample consists of families with children aged 6 months to 47 months at t1 (N = 200). The risk group (N = 100) includes families where at least one biological parent is obese (BMI ≥ 30 kg/m^2^). The comparison group (N = 100) consists of families where both parents are normal weight (18.5 ≤ BMI < 25 kg/m^2^). The two groups should preferably be matched according to age and sex of the child and the social economic status of the family.

The sample size (100 subjects in the risk group and 100 subjects in the comparison group) will have sufficient power for group comparisons such as those proposed in our research objectives (e.g. point 2). Our sample size will have a power of > .90 to detect small to medium effect sizes in a two-sided difference test with a type I error level of 0.05 (calculated using G-Power®, [80,81]). Here, effect sizes are classified according to Cohen’s recommendation of the effect size d for t-tests with d = .2 considered small, d = .5 medium, and d = .8 large effects [82].

For ensuring adherence to the longitudinal study protocol, the families receive a financial incentive (€25) and a small gift for the child at each assessment point. Furthermore, we offer the families high flexibility in time and date of appointments. To minimize the drop-out rate, we send families information about first results, a sticker with the corporate study design as well as Christmas cards and offer e.g. lectures about nutrition and feeding.

### The IFB AdiposityDiseases Leipzig

The presented research study is part of the Integrated Research and Treatment Center (IFB) AdiposityDiseases Leipzig. It is one of eight Integrated Research and Treatment Centers in Germany funded by the Federal Ministry of Education and Research (BMBF). The aim is an interdisciplinary link of research and treatment to integrate scientific findings as fast as possible in the clinical process. The IFB AdiposityDiseases Leipzig includes 40 research projects which investigate different issues related to obesity. An international External Advisory Board evaluates the research projects and the structural design of the Integrated Research and Treatment Center.

### Ethics approval

The study has been approved by the ethics committee of the University of Leipzig, Germany (registration number 039-09/09032009). Parents are informed orally and in written form about the contents and aims of the study and have to give their written consent. All participation is voluntary. None of the investigations is in any way dangerous and no risk is associated with any tests used.

### Data assessment

At each assessment point, we assess data on three levels: the child, the parents, and the family. On the level of the child, we collect data regarding their physical and psychological development either using age appropriate tests and procedures or by parent-report. To assess data from a person outside of the family we use reports of preschool teachers. On the level of the parents, we assess physical variables as well as their psychological situation. For this purpose, parents are interviewed and they fill in questionnaires. On the level of the family, we measure parents’ interaction with the child in a play and a feeding situation which is video-taped in our research laboratory.

### Instruments

In the following, variables and instruments are specified and shortly described, beginning with (a) the level of the child’s individual psychological and biological functioning. Table 1 gives an overview and indicates at which assessment point the variables are assessed.

**Table 1:** Assessment of child’s individual psychological and biological functioning

**Variable**	**Method of data collection**	**Assessment point**		
		t1	t2	t3
Height and weight	Measurement in the lab	x	x	x
Physical development	Copy from the “U-Heft”	x	x	x
Length of sleep	Self-generated questions	x	x	x
Quality of sleep	German version of Children’s Sleep Habits Questionnaire (CSHQ-DE)			x
Temperament	Infant Behavior Questionnaire Revised (IBQ-R)	x		x
	Early Childhood Behavior Questionnaire (ECBQ)	x		x
	Children’s Behavior Questionnaire (CBQ)	x		x
Psychological symptoms	Interview based on criteria of the diagnostic classification of mental health and developmental disorders of infancy and early childhood ZERO TO THREE (revised)	x	x	x
	Child Behavior Checklist (CBCL/1.5–5), parents version	x	x	x
	Caregiver-Teacher Report Form (C-TRF/1.5–5)		x	x
Peer relationship problems and Prosocial behavior	Corresponding scales of the Strength and Difficulties Questionnaire (SDQ), for parents and kindergarten teachers		x	X
Social-emotional competence	Corresponding scale of the German questionnaire „Verhaltensbeurteilungsbogen für Vorschulkinder“ (VBV 3–6), for parents and kindergarten teachers		x	x
Emotion understanding	Test of emotion comprehension		x	
Eating behavior	Children’s Eating Behaviour Questionnaire (CEBQ)		x	x
Early nutrition	Questionnaire with self-generated questions	x	x	x
Activity, media use, lifestyle	Questionnaire with self-generated questions from the PEB study		x	x
Behavioral regulation	Head-Toes-Knees-Shoulders Task (HTKS)			x
Delay of gratification	Marshmallow Test			x

In order to assess the physical development of the child we measure the *height and weight* at every assessment point and derive BMI percentiles. The *physical development* of height, weight and BMI percentiles since birth is taken out of the “U-Heft”. The parents of each child in Germany keep this official booklet including the routine medical check-ups U1 (at birth), U2 (3^rd^ until 10^th^ day), U3 (4^th^ until 5^th^ week), U4 (3^rd^ until 4^th^ month), U5 (6^th^ until 7^th^ month), U6 (10^th^ until 12^th^ month), U7 (21^st^ until 24^th^ month) and U7a (34^th^ until 36^th^ month). The *length of sleep* is assessed by parent report. The German version of the Children’s Sleep Habits Questionnaire (CSHQ-DE) is used to measure the *quality of sleep*[83,84]. To measure the *temperament* of the child as one of the assessed psychological variables, we use the very short forms of the Infant Behavior Questionnaire Revised, IBQ-R [85], the Early Childhood Behavior Questionnaire, ECBQ [86] and the Children’s Behavior Questionnaire, CBQ [87] depending on the age of the infant. Parents are asked to report on specific behaviors during specific events during the last week (IBQ-R), the last two weeks (ECBQ) or the last 6 months (CBQ). Factor analyses of ECBQ and CBQ revealed a three-factor structure: Extraversion/Surgency, Negative Affectivity, and Effortful Control. For the IBQ-R a three factor solution emerged as well with the dimensions of Positive Emotionality/Surgency, Negative Affectivity, and Orienting/Regulatory Capacity. An interview with the parents based on the criteria of the revised version of the diagnostic classification of mental health and developmental disorders of infancy and early childhood ZERO TO THREE is used to assess regulation disorders, sleep behavior disorders, feeding behavior disorders, depression, anxiety and other *psychological symptoms*[88]. Furthermore, in order to assess behavioral problems in the children, we ask their parents and kindergarten teachers to fill in the Child Behavior Checklist (CBCL/1.5–5) by Achenbach and Rescorla [89] for children aged 18 months to 5 years. In this revision of the 1992 checklist for children aged 2–3 years [90], 99 specific child behaviors are rated on a 3 point Likert scale (0 = not true, 1 = somewhat or sometimes true, or 2 = very true or often true). We additionally ask parents and kindergarten teachers to report on children’s *peer relationship problems and prosocial behavior* by using the homonymous scales of the Strength and Difficulties Questionnaire [91,92] and the *social-emotional competence* with the corresponding scale of the German questionnaire VBV 3–6 [93]. We also test children’s *emotion understanding* with the Test of Emotion Comprehension [94,95]. The child’s *eating behavior* is assessed with the Children’s Eating Behaviour Questionnaire (CEBQ), a parent-rated instrument measuring eight dimensions of eating style in children [96]. Included dimensions are responsiveness to food, enjoyment of food, satiety responsiveness, slowness in eating, fussiness, emotional overeating, emotional undereating and desire for drinks. Additionally, we obtain parental reports on the *early nutrition* of the child including the duration of breastfeeding, the time of weaning, the duration of bottle feeding, and the time of introducing solid foods as well as on the child’s *activities, media use, and lifestyle*. We use a questionnaire with self-generated questions from the PEB study [97]. Furthermore, we assess the child’s *behavioral regulation*, including inhibitory control, attention, and working memory, with the Head-Toes-Knees-Shoulders (HTKS) task [98]. The HTKS was developed as a more complex and extended version of the Head-to-Toes task [99,100] which asks children to perform the opposite of a dominant response to four different oral commands (“touch your head”, “touch your toes”, “touch your knees” and “touch your shoulders”). Child’s ability to *delay gratification* is measured with the Marshmallow Test by Walter Mischel [101]. We use a modified Marshmallow Test: The experimenter presents a desired chocolate waffle to the child. Then the experimenter informs the child that he/she will get an additional chocolate waffle if he/she waits to eat until the experimenter returns. The experimenter leaves the room and observes the child through a mirror and returns after the child has taken the waffle, but not later than after15 minutes with the additional chocolate waffle. The child’s behavior is video-taped. Afterwards times of delay until eating, looking, touching, and licking the object are measured.

On (b) the level of the parental functioning (see Table 2) we measure parents’ *height and weight* (for mothers at the earliest at 6 months after birth to allow weight stabilization). *Mothers’ weight before and during pregnancy* and health *problems during pregnancy* including the occurrence of gestational diabetes or complications are assessed by using the expectant mothers’ record of prenatal and natal care (“Mutterpass”). To assess parents’ *attitudes to feeding* the Maternal Feeding Attitudes Scale (MFA) is used [102]. The scale (10 items) was developed in order to “measure psychological factors that may affect infant feeding and thus “confound” the relationship between feeding and subsequent obesity“ [102]. Furthermore, a screening for *mental disorders* and psychosocial stressors is performed with the German version of the Patient Health Questionnaire (PHQ), which refers to diagnostic criteria from the DSM-IV [103]. In addition, we assess the *body-image* of the parents with the Multidimensional Body Self-Relations Questionnaire, which includes affective, cognitive, and behavioral components [104]. The 34-item version consists of five subscales: Appearance Evaluation, Appearance Orientation, Body Areas Satisfaction, Overweight Preoccupation, and Self-Classified Weight. In order to assess *parenting stress*, we use the short form of the Parenting Stress Index, Third Edition (PSI:3) which is a 36-item questionnaire consisting of three scales: Parental Distress, Parent–child Dysfunctional Interaction, and Difficult Child [105]. The German items were taken from the German version of the PSI by Hofecker and colleagues [106]. To assess the parental *lifestyle*, we adapted the Leipziger Lebensstil-Fragebogen für Jugendliche (LLfJ) [107] which is a lifestyle questionnaire for adolescents in order to use it for adults. Parents also report on their *parenting practices*. For this purpose we use the adapted German version of the Parenting Practices Questionnaire [108,109] which consists of three dimensions (authoritarian, authoritative, permissive). Furthermore, parents report on their *satisfaction with life* by filling in the Satisfaction with Life Scale (SWLS), a self-report questionnaire including five items [110], and on their self-esteem by answering the 10 items of the Rosenberg Self-Esteem Scale (RSE) which is the most used, analyzed, and empirically validated instrument to assess *global self-esteem*[111,112]. The assessment of parental *eating style* is performed with the Dutch Eating Behaviour Questionnaire (DEBQ) which contains 33 items measuring three subscales: Restrained Eating with 10 items, Emotional Eating with 13 items, and External Eating with 10 items [113]. A revised German version is available [114]. Additionally, we measure the parental *feeding style* with a questionnaire by Kröller and Warschburger (2009) [115] which is besides self-constructed items based on translated items of the Child Feeding Questionnaire (CFQ) [116] and the Caregiver's Feeding Styles Questionnaire (CFSQ) [117]. The 6 following factors were derived: restriction monitoring, pressure, rewarding, child’s control, and modelling [115,118]. To assess aspects of parents’ *emotion recognition and understanding*, we use the German version of the Toronto Alexithymia Scale [119,120] which is a self-report questionnaire consisting of three subscales: Difficulty identifying feelings, Difficulty describing feelings, and Externally oriented thinking. Furthermore, we use the subscales C and G of the German version of the Mayer-Salovey-Caruso Emotional Intelligence Test (MSCEIT, [121,122]) as well as an experimental task for decoding facial expressions of emotions using pictures from the Radboud Faces Database [123]. Additionally, we assess parental perception and interpretation of infants’ emotion by using the Infant Facial Expressions of Emotion from Looking at Pictures (IFEEL Pictures, [124]). Moreover, an interview with the mothers gives information about mothers’ mentalization *(mothers’ reflective functioning)* quality. Therefore, we use the semistructered Adult Attachment Interview [125] and the coding system by Fonagy and colleagues [126].

**Table 2:** Assessment of parental functioning

**Variable**	**Method of data collection**	**Assessment point**		
		t1	t2	t3
Height and weight	Measurement in the lab	x	x	x
Mothers’ weight before and during pregnancy, problems during pregnancy	Copy from the “Mutterpass”	x		
Attitudes to feeding	Maternal Feeding Attitudes Scale (MFA)	x		
Mental disorders	German version of the Patient Health Questionnaire (PHQ)	x	x	x
Body-image	Multidimensional Body Self-Relations Questionnaire			x
Stress	Short form of the Parenting Stress Index, Third Edition (PSI:3)	x	x	x
Lifestyle	adapted version of the Leipziger Lifestyle questionnaire for adolescents (LLfJ)	x		x
Parenting practices	Adapted German version of the Parenting Practices Questionnaire		x	x
Satisfaction with Life	Satisfaction with Life Scale (SWLS)		x	x
Global self-esteem	Rosenberg Self-Esteem Scale (RSE)			x
Eating style	Dutch Eating Behaviour Questionnaire (DEBQ)		x	x
Feeding style	German Questionnaire “Instrument zur Erfassung elterlicherSteuerungsstrategien in der Essenssituation” (ISS), based on the Child Feeding Questionnaire (CFQ) and Caregiver's Feeding Styles Questionnaire (CFSQ)		x	x
	Caregiver's Feeding Styles Questionnaire (CFSQ)			
Emotion recognition and understanding	German version of the Toronto Alexithymia Scale (TAS-26)	x		
	German version of the MSCEIT (Subscales C and G)	x		
	Decoding facial expressions of emotions	x		
	Infant Facial Expressions of Emotion from Looking at Pictures (IFEEL)	x		
Mothers’ reflective functioning	Adult attachment interview with the coding system by Fonagy and colleagues		x	

On (c) the level of the family and parent–child interaction (see Table 3) *socioeconomic status* with the categories “Low”, “Middle”, and “High” is assessed with the Winkler Index for social classes. The index takes into account parents’ education, professional training, profession, and household income [127]. Furthermore, mothers and fathers rate the *familial relationships* using the corresponding dimension of the German adaption of the Family Environment Scale [128-130]. It applies 18 items which constitute the subscales Cohesion, Expressiveness, and Conflict. The mother-child as well as the father-child *interactions during feeding* is video-taped and rated with the Feeding Scale by Chatoor and colleagues [131]. This scale can be used for infants and toddlers from 1 month to 3 years and consists of the following subscales: Dyadic Reciprocity, Dyadic Conflict, Talk and Distraction, Struggle for Control, and Maternal Non-Contingency. To assess *emotional availability*, the interactions of mothers and fathers with their children during playing are video-taped and rated using the Emotional Availability Scales, 4^th^ edition [132,133]. These scales assess six dimensions, four on the adult side: Sensitivity, Structuring, Nonintrusiveness, and Nonhostility as well as two on the child side: Responsiveness to adult and Involvement of adult. On top of that we evaluate *mother-child-attachment*. A subsample of mothers with their children is observed for two hours at home. Afterwards, the trained observer completes the 90 items of the Attachment Q-Sort (AQS, [134]) about the child’s behavior.

**Table 3:** Assessment of family variables and parent–child interaction

**Variable**	**Method of data collection**	**Assessment point**		
		t1	t2	t3
Socioeconomic status	Winkler index	x	x	x
Familial relationships	German adaption of the family environment scales	x	x	x
Feeding interactions	Chatoor feeding scale	x		
Emotional availability	Emotional availability scales, 4^th^ edition	x		
Mother-child-attachment	Attachment Q-Sort (AQS) after observing at home for two hours		x	

### Statistical analyses

We will test cumulative and mediating models. Cumulative means that, for example, parental obesity and parental stress are additive risk factors in their effect on the child’s weight development. On the other hand, mediation would mean that one risk factor (e.g. parental obesity) leads to another factor (e.g. a certain parental interactional behavior) which then leads to children’s obesity.

For *confirmatory analyses*, group comparisons between obese and normal weight parents will be analyzed using Likelihood Ratio Chi^2^-tests for dichotomous or categorical variables and t-tests for continuous variables. Nonparametric tests such as Mann–Whitney U-tests may be applied if variables are not normally distributed. Risk factor models will be built using logistic regressions. The main effect model will be specified first, and the importance of additional factors will be evaluated subsequently by comparison with the main factor model and tests of improvement of model fit. Odds ratios and measures of effect sizes will be computed. Structural equation modelling techniques [135] will be applied to investigate the mediating mechanisms specified above. Path models with and without the mediating factors will be evaluated and an integrated multivariate model will be built and tested.

In addition to the confirmatory analyses, the longitudinal data set with its comprehensive assessments on the physical, psychological, family and behavioral (video) level of parents and children provides a unique opportunity for *exploratory and hypotheses generating analyses*. In particular, we are interested in correlates and predictors, risk and protective factors for childhood overweight/obesity beyond those expressed in a priori hypotheses. We will perform three subsets of analyses: first, comparing four groups that are defined by all possible combinations of overweight/obese and normal weight children and parents, second, comparing overweight/obese vs. normal weight children within children with obese parents, and third, comparing children with one vs. two obese parents. However, sample size in any of these subsets may be too small to perform statistical tests and to provide p-values. In this case, summary statistics such as means and standard deviations, medians and quartile ranges, or proportions with confidence intervals will be calculated to describe the data. If sample size is sufficient, to test for group differences, analyses of variance (or nonparametric Kruskal-Wallis H-tests) or t-tests (or nonparametric Mann–Whitney U-tests) will be used. Here, special attention will be paid to account for multiple testing. Appropriate techniques include Bonferroni adjustments or methods developed by Holm [136]. To explore multivariate models, logistic or linear regressions will be performed. Factor analyses will be applied to reduce the number of potential predictors prior to building regression models.

## Discussion

The main strength of the planned study is the prospective longitudinal investigation of physical and psychological development of young children at overweight risk taking into account risk and protective factors on the level of the child, the parents and the family. The multi-informant approach is another strength. We assess data from mothers, fathers, and preschool teachers. Besides mothers, fathers give information on familial conditions which is rarely studied so far. Moreover, observational data permits an objective measurement of parents’ interaction with the child in a play and a feeding situation that goes beyond parents’ evaluations. The longitudinal study design allows cross-sectional analyses and statements after each assessment point as well as the analysis of the developmental trajectories and evaluation of the risk factor which predict the development of overweight and obesity after the third assessment point.

There are also limitations to the study. First, the children in the sample are not all of the same age – they are between 6 and 47 months at the first assessment point. Therefore, we need to take age into account when interpreting the results. However, as the study with the three assessment points (approximately one year apart) extends over a relatively long period, we assess children from 6 months to 5;11 years; months which is significant in terms of development of the children. Second, the families of the risk group are recruited because they have at least one obese parent. This may result in very different family constellations; for instance, it could be the mother or the father who is obese, both parents could be obese, or one of them overweight, each constellation may have different consequences for the child’s weight or mental development. Depending on the resulting distribution of subgroups, the sample size might be too small to test for all subgroup differences. Third, prior research has identified a number of barriers in recruiting families for obesity clinical trials [137,138] and willingness to participate in obesity studies seems to be low [139,140]. Therefore, it is challenging to avoid a self-selection bias.

The clinical implication of our study is that the results and the risk factors identified might provide a rationale for the creation of an early prevention or treatment program for obese parents with their infants and toddlers.

## Competing interests

The authors declare that they have no competing interests.

## Authors’ contributions

All authors have made substantive intellectual contributions to this project. KvK is the principal investigator and has overall responsibility for the study. He conceived and designed the study with the collaborating investigators AMK and MG. They guided the development of the study protocol until presentation to the international External Advisory Board, responded to reviewer’s comments and worked out the successful ethics proposal. VW and SB have been involved in the development of the study from conception to current practice and were as well as AK and KHM actively involved in coordination, recruitment and establishment of the cohort. AK was responsible for those parts of the study regarding attachment and mentalization measures. All authors contributed to the manuscript of the study protocol and approved the final manuscript.

## Pre-publication history

The pre-publication history for this paper can be accessed here:

http://www.biomedcentral.com/1471-2458/13/1193/prepub
